# Direct Evidence of Salinity and pH Effects on the Interfacial Interactions of Asphaltene-Brine-Silica Systems

**DOI:** 10.3390/molecules25051214

**Published:** 2020-03-08

**Authors:** Fanghui Liu, Hui Yang, Ting Chen, Shanmeiyu Zhang, Danfeng Yu, Yongqiang Chen, Quan Xie

**Affiliations:** 1 CAS Key Lab of Colloid, Interface and Chemical Thermodynamics, Institute of Chemistry, Chinese Academy of Sciences, Beijing 100190, China; liufanghui@iccas.ac.cn (F.L.); chenting417@iccas.ac.cn (T.C.); zhangsmy@iccas.ac.cn (S.Z.); 2 University of Chinese Academy of Sciences, Beijing 100049, China; 3 School of Chemistry and Chemical Engineering, Guangzhou University, Guangzhou 510006, China; ccyudanfeng@gzhu.edu.cn; 4 Department of Petroleum Engineering, Curtin University, Kensington 6151, Western Australia, Australia; yongqiang.chen@postgrad.curtin.edu.au

**Keywords:** salinity and pH effects, interfacial interactions, asphaltene adsorption/deposition, surface wettability, surface complexation modelling

## Abstract

The hydraulic fracturing technique remains essential to unlock fossil fuel from shale oil reservoirs. However, water imbibed by shale during hydraulic fracturing triggers environmental and technical challenges due to the low flowback water recovery. While it appears that the imbibition of fracturing fluid is a complex function of physico-chemical processes in particular capillary force which is associated with wettability of oil-brine-shale, the controlling factor(s) to govern the wettability is incomplete and the literature data in this context is missing. We thus measured the adsorption/desorption of asphaltenes on silica surface in the presence of brines using quartz crystal microbalance with dissipation (QCM-D). We detected zeta potential of asphaltene-brine and brine-silica systems and calculated the disjoining pressures of the asphaltene-brine-silica system in the case of different salinity. Moreover, we performed a geochemical study to quantify the variation of surface chemical species at asphaltene and silica surfaces with different pH values and used the chemical force microscope (CFM) method to quantify the effect of pH on intermolecular forces. Our results show that lowering salinity or raising pH reduced the adhesion force between asphaltene and silica surface. For example, at a pH value of 6.5, when the concentration of injected water is reduced from 1000 mM to 100 mM and 10 mM, the adhesion force decreased by approximately 58% and 66%, respectively. In addition, for the 100 mM NaCl solution, when the pH value increased from 4.5 to 6.5 and 9, the adhesion force decreased by approximately 56% and 87%, respectively. Decreased adhesion forces between asphaltene and the silica surface could promote the desorption of asphaltene from the silica surface, resulting in a negative zeta potential for both asphaltene-silica and brine-silica interfaces and a shift of wettability towards water-wet characteristic. During such a process, -NH^+^ number at asphaltene surfaces decreases and the bonds between -NH^+^ and >SiO^−^ break down, to further interpret the formation of a thinner asphaltene adlayer on the rock surface. This study proposes a reliable theoretical basis for the application of hydraulic fracturing technology, and a facile and possible manipulation strategy to increase flowback water from unconventional reservoirs.

## 1. Introduction

Low emission energy is an indispensable resource in modern society and, therefore, both crude oil and natural gas resources are rapidly expanding to cope with the global growing energy needs [1,2]. The decline in conventional oil reserves has aroused great attention and transferred widespread interest into unconventional reservoirs, such as low permeability in a range of 1 to 1000 nano-darcy [3]. To economically unlock the oil and gas resources from such reservoirs, hydraulic fracturing needs to be implemented to extend the fracture network and activate new fractures, resulting in the enhancement of connectivity. However, ~70% of fracturing fluids are often difficult to recover during a hydraulic fracturing process [3,4], especially for the asphaltene-rich shale reservoirs, which received widespread attention in both technical and environmental fields. Various work has been implemented to understand why shale reservoirs yield low flowback water recovery during hydraulic fracturing, e.g., the imbibition of fracturing fluid on shale matrix and micro-fractures [5,6,7], the hydration of clay minerals [8,9], and the reduced permeability [10,11,12]. A generally accepted theory believed that during fracturing fluid injection, water will not imbibe into the hydrophobic matrix but will preferentially enter the hydrophilic micro-fractures and result in a low fracturing fluid recovery [13,14,15]. Thus, the oil recovery in low permeability oil reservoirs depends critically on the wetting properties of the shale matrix. It is important to explore the wettability alteration mechanism in improving oil recovery, especially to find an effective method to improve the wetting property of reservoir surfaces. In previous reports, a series of contact angles on shale samples were measured with aqueous ionic solutions at different concentrations, indicating that the shale had a higher affinity for more diluted solutions [16]. Some other experimental results show that the presence of divalent cations can significantly increase surface wettability compared to monovalent cations [2,17]. In addition, there are related studies claiming that ion type and strength significantly affect the wettability of the oil-brine-rock system, but the results were mostly obtained from macroscopic changes such as contact angle and interfacial tension [18,19,20]. Therefore, quantitative research and responsible mechanisms for wettability alteration are a great need.

To provide such a framework, we examined the adsorption/desorption of asphaltene on silica surface in the presence of NaCl aqueous solutions at a series of concentrations using the quartz crystal microbalance with dissipation (QCM-D) method and investigated the role of water uptake during these processes as well as wettability alteration. Based on such QCM-D results and our previous analysis of asphaltene desorption [18], we also used chemical force microscope (CFM) technology to directly measure the interaction between asphaltene and silica surface, and combined it with the Derjaguin-Landau-Verwey-Overbeek (DLVO) theory and surface complexation modelling to further delineate the interfacial interactions.

## 2. Results and Discussion

### 2.1. Effect of Salinity on Adsorption/Desorption of Asphaltene on Silica Surface

**Figure 1:**
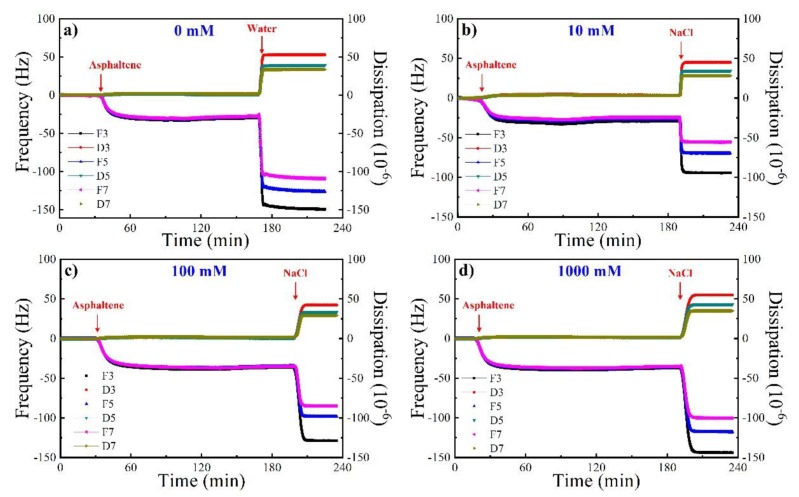
Frequency and dissipation shift as a function of time during asphaltene adsorption and (**a**) 0 mM, (**b**) 10 mM, (**c**) 100 mM, (**d**) 1000 mM NaCl rinsing.

Figure 1 shows the frequency and dissipation variation during the asphaltene injection, pure solvent rinsing, and brine injection. F3, F5, F7 and D3, D5, D7 represent the frequency and dissipation curves corresponding to different overtones, respectively. When the frequencies and dissipation curves under different overtones coincide, it means that a rigid adsorption layer is formed. On the other hand, if they do not overlap, it means that the formed adsorption layer is loose. Resonance frequency represents the information of the adsorbed mass on surface, and energy dissipation provides the information of the elastic property of the adsorbed film, from which frequency decrease means asphaltene adsorption and dissipation increase means loose adsorption conformation. [21,22]. A decrease of 25–35 Hz in frequency and an increase of 2–3 × 10^−6^ in dissipation are obtained due to the asphaltene adsorption in all cases, indicating the reproducibility and credibility. Negligible variations of frequency and dissipation values at different overtones are also found during the injection of asphaltene, indicating the formation of rigid films in line with previous reports [12,23]. After the adsorption equilibrium, the surface is rinsed by pure solvent toluene, and then the aqueous solution is introduced which triggers sharp changes in both frequency and dissipation, in the formation of swelling adlayers. This is largely due to the liquid loading effect as a result of differences in viscosity and density between toluene and water [24].

With the addition of the NaCl solution, the frequency decreases from the range of −50 to −90 Hz to −100 to −150 Hz with the salinity increasing from 10 mM to 1000 mM, whereas the dissipation has a slow rising trend around 50 × 10^−6^ with increasing salinity. Compared with pure water, the frequency is higher in the presence of 10 mM NaCl than that in the pure water system, similar in the presence of 100 mM NaCl with that in the pure water system, and lower in the presence of 1000 mM NaCl than that in the pure water system (Figure 1a). These results imply that, on the premise of the same liquid loading effect in all the aqueous solutions, lowering salinity facilitates the desorption of asphaltene, suggesting that salinity has a significant impact on the asphaltene-brine-silica interaction. We speculate that this is attributed to the compaction of electrical double layer between asphaltene and quartz as a result of increasing salinity. Therefore, more asphaltene can be removed at lower concentrations of NaCl, and after the desorption of more polar sites on the substrate would be available for water molecules, triggering a more water-wet surface.

### 2.2. Effect of Salinity on Zeta Potential of Asphaltene-Brine-Silica System

Decreasing salinity results in more negative zeta potentials for both asphaltene-brine and brine-silica interfaces, suggesting an increase of repulsive forces with decreasing salinity, thus a more water-wet system. For example, zeta potential decreases from −6.6 to −17.1 mV as salinity decreases from 1000 to 10 mM for asphaltene-brine (Table 1). Similarly, zeta potential decreases from −1.5 to −29.0 mV as salinity declines from 1000 to 10 mM for brine-silica (Table 1). Similar results were also reported by research groups, they claimed that zeta potential of brine-kaolinite systems decreased from −10 to −30 mV with decreasing salinity from 50 to 2000 mg/L of NaCl [25]. Likewise, zeta potential of brine-mineral and brine-oil follows the same trend as brine-kaolinite in the presence of various brines. That is, decreasing the salinity leads to more negative zeta potentials at both asphaltene-brine and brine-silica interfaces, and reduces the expansion of the electrical double layer and decreases the repulsive forces, which results in a more water-wet state and facilitates asphaltene desorption from the surface. This also explains why more asphaltenes can be removed from the silica surface at lower NaCl concentrations in the above QCM-D experiment.

### 2.3. Effect of Salinity on Interactions of Asphaltene-Brine-Silica System

**Figure 2:**
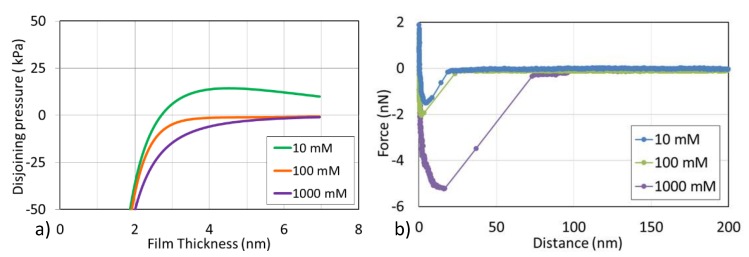
(**a**) Disjoining pressures and (**b**) Adhesion forces of asphaltene-brine-silica systems in the presence of various salinities.

Here, disjoining pressure was used to characterize the interaction forces in the asphaltene-brine-silica system. Positive disjoining pressure means repulsive forces between asphaltene and silica in the brine, suggesting a relatively water-wet system, whereas negative disjoining pressure means attractive forces between asphaltene and silica in the brine, implying a relatively oil-wet system [26]. Our calculations indicate that with the decrease in brine concentration, disjoining pressure changes from negative to positive, which implies that lowering salinity likely shifts an oil-wet system to a water-wet system (Figure 2a). For example, in the presence of 1000 mM and 100 mM NaCl, disjoining pressure is negative and monotonically decreases with decreasing separation distance. For 10 mM NaCl, the disjoining pressure exhibits a positive value indicating the presence of repulsion. It is worth noting that the decrease in salinity triggers the outward expansion of the electrostatic double layer, resulting in an increase in electrostatic repulsion [27]. The disjoining pressure calculation reasonably explains the above zeta potential experimental results, implying that lowering salinity induces more negative zeta potential of asphaltene-brine and brine-silica interfaces, resulting in the repulsive force and water-wet system.

In addition, we have directly measured the interaction between the asphaltene model compound and surfaces by the aid of a chemical force microscope (Figure 2b). The adhesion forces between asphaltene and surfaces were obtained by the max deflection on the y axis from every retract force curve. For example, at a concentration of 10 mM, the adhesion force was only about 1.5 nN, and at 1000 mM, the adhesion force increased to 5.0 nN. It can be considered that the adhesion force increased as the salinity increased. These results are also in line with the calculations data of disjoining pressure (Figure 2a), showing that as the salinity decreased from 1000 to 10 mM, the adhesion forces between asphaltene and surface are significantly reduced, which is beneficial to the desorption of asphaltene from silicon surface. It is worth noting that this is also the reason for low salinity NaCl solution peeling off more asphaltene.

Moreover, the interaction of the asphaltene-brine-silica system is also thought to be governed by thermodynamics [26,27,28,29], which can be depicted using DLVO theory, together with electrostatics, [23,30,31,32], which can be described using surface complexation modelling. In order to understand how the surface species at asphaltene-brine and brine-silica interfaces impact the interaction of asphaltene-brine-silica system, a geochemical study was performed in the following subsection.

### 2.4. Effect of Salinity/pH on Interactions of Asphaltene-Brine-Silica System

**Figure 3:**
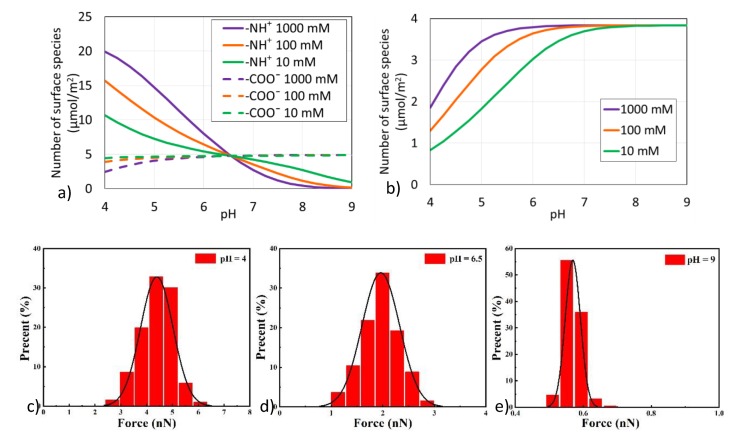
Effect of pH on (**a**) Number of surface species at asphaltene surfaces and (**b**) Number of surface species at silica surfaces in the presence of various salinities, and Adhesion forces of asphaltene-brine-silica systems in the presence of 100 mM NaCl at (**c**) pH 4, (**d**) pH 6.5, and (**e**) pH 9.

From surface complexation modelling, it indicates that −NH^+^ dominates asphaltene surface charges at low pH (pH < 6) in line with previous reports [33]. The number of -NH^+^ declines with decreasing salinity as pH < 6.5, whereas the opposite trend is observed, showing that the number of −NH^+^ increases with decreasing salinity as pH > 6.5 (Figure 3a). For example, at pH = 5, −NH^+^ decreases from 14.8 to 7.2 µmol/m^2^ as salinity declines from 1000 to 10 mM. A similar trend has been observed before, indicating that when pH < 6.5, the number of -NH^+^ groups increase as the ionic strength increases; when pH > 6.5 the number of −NH^+^ groups on asphaltene surface decreases as the ionic strength increases [33]. Compared with −NH^+^, salinity has a minor effect on the number of −COO^−^ at a given pH, showing that increasing salinity decreases the number of −COO^−^, due to the fact that asphaltene has a low acid number (2.75 mg KOH/g) but high base number (12.34 mg KOH/g). For example, at pH = 5, increasing salinity from 10 to 1000 mM decreases the number of −COO^−^ only from 4.7 to 4.0 µmol/m^2^. Without adjusting the pH value, the numbers of −NH^+^ and −COO^−^ in our brine systems during the pH value around 6.5 are similar and, therefore, there is little effect of salinity on the number of surface species at asphaltene-brine interfaces.

Increasing salinity increases the number of >SiO^−^ at the silica surfaces at pH < 7, implying more bonds between SiO^−^ and -NH^+^ and resulting in a more oil-wet system. For example, at pH = 5, >SiO^−^ increases from 1.8 to 3.5 µmol/m^2^ (Figure 3b). It is also found that increasing pH, in particular pH > 7, triggers a negligible effect of salinity on the number of surface species. For example, at pH = 7, >SiO^−^ increases from 3.7 to 3.8 µmol/m^2^ as salinity increases from 10 to 1000 mM. In our given pH condition around 6.5, the number of >SiO^−^ increases as salinity increases from 10 to 1000 mM, which may result in more >SiOH sites and a bigger bond product sum between asphaltene-brine and brine-silica interfaces. Accordingly, the attractive interactions between interfaces are likely to be enhanced with the increase of salinity, inducing more asphaltene attached to the silica surface and more oil-wet.

In order to verify the pH effect shown in the calculation results of the above surface complexation modelling, we measured the adhesion forces between the asphaltene model compound and silica surface at different pH conditions and plotted statistical graphs for easy observation. Because the effect of pH condition is consistent at each concentration, here only 100 mM NaCl solution was used as an example for discussion. At pH = 4, the adhesion forces between asphaltene model compound and silica surface is about 4.5 nN (Figure 3c), indicating that asphaltene has a strong adhesion tendency to the silica surface under low pH conditions. When pH = 6.5, the adhesion forces decreases to around 2.0 nN (Figure 3d), and at pH = 9, the adhesion is almost zero (Figure 3e), showing that an increase in pH would weaken the adhesion forces between asphaltene and silica surface. Therefore, as the pH value increases, the attractive interactions between the interfaces are likely to be weakened, resulting in more asphaltenes desorbed from the silica surface and increasing the hydrophilicity of the rock surface.

## 3. Materials and Methods

### 3.1. Experiments

#### 3.1.1. Asphaltene Extraction and Characterization

**Figure 4:**
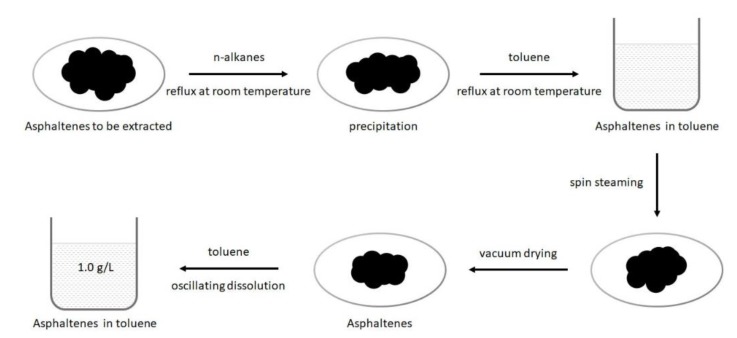
Schematic diagram of the asphaltenes extraction process.

Given that unconventional reservoirs are rich in organic matters [34,35,36], and asphaltene is widely distributed at pore surfaces [37,38,39], asphaltene was used in this work to represent the organic matters in unconventional reservoirs. The experimental asphaltenes was extracted from oil sand (Long Lake, Ontario, Canada) as the adsorbate for quartz crystal microbalance experiments. The asphaltenes extraction process is detailed in our previous publication [17], and the main steps are shown in Figure 4.

#### 3.1.2. Brines

NaCl aqueous solutions at three concentrations of 10, 100, and 1000 mM were used in the experiments. NaCl (AR, 99.5%) with Milli-Q grade water with a resistivity of 18.2 MΩ·cm at the concentration of 1000 mM was used to deploy the experimental brines. The initial pH values of 10 mM, 100 mM, and 1000 mM NaCl solutions were about 7.09, 6.76, and 6.13, respectively. When exploring the salinity effect, all the pH values of the solutions were adjusted to 6.5 ± 0.05 using HCl or NaOH to get rid of the pH effect on the adsorption of asphaltene on the silica surface. When exploring the pH effect, 4, 6.5, and 9 were chosen to represent acidic, neutral and alkaline solutions, respectively. All the solution preparations and pH adjustment operations are performed at 25 °C.

#### 3.1.3. Experimental Procedure

QCM-D (Q-SENSE E4, Biolin Scientific, Stockholm, Sweden) was used to measure the adsorption and desorption of asphaltene on silica-coated quartz crystal sensors (QSX 303, Biolin Scientific, Sweden) in the presence of various brines. In the experiment, the adsorption/desorption behavior of asphaltene on the silica surface was monitored in real time by recording the frequency and dissipation curve over time. Frequency shift is used to indicate the change in mass on the surface. A negative shift indicates that molecules are adsorbed on the surface, and a positive shift indicates that molecules are removed from the surface. Dissipation is used to characterize the softness of the adsorption layer on the surface. For example, large dissipation indicates loose molecular arrangement. Different harmonics can be collected, and in this work three of them are selected for analysis. Here we briefly described the procedures of the experiments because the principle and the detailed experimental procedures of QCM-D experiments were documented in our previous work [18]. Silica crystals’ surfaces were cleaned with 5 wt% sodium dodecyl sulfate (SDS) solution for 60 min, followed by thoroughly rinsing three times with ethanol (AR, 99%) and Milli-Q water. After the acquirement of baseline in air, the experiments are divided into four steps: 1) Toluene is introduced as the background solution until the baseline is flat; 2) The asphaltene is introduced and then a significant decrease in frequency appears; 3) The background solution (toluene) is introduced again to remove the weakly bound components; 4) Brine solutions at 10, 100, and 1000 mM were introduced respectively to measure the desorption process of asphaltenes from solid surfaces. All the adsorption/desorption experiments were executed with a constant flow rate of 50 μL min^−1^.

Atomic force microscopy (Multimode VIII, Bruker Instruments, Santa Barbara, CA, USA) was used to measure the adhesion forces between asphaltene and silica surfaces at the molecular level. *N*-(1-hexylheptyl)-*N*′-(5-carboxylicdodecyl) perylene-3,4,9,10-tetracarboxylic bisimide (C5Pe) was selected as a model compound to mimic asphaltene. The method of using model compounds to establish the relationship between the asphaltene molecular structure and interface properties is described in detail in our previous work [40]. The following is a brief introduction for the experimental process: 1) Functionalization tips were prepared by modifying the C5Pe molecules to gold-plated commercial atomic force microscope (AFM) tips (NPG-10, Bruker Instruments, Santa Barbara, USA); 2) silica as a substrate to simulate the surface of reservoirs; 3) the interaction between C5Pe molecules and silica surface were measured with the contact mode by AFM in different NaCl environments; 4) adhesion curves were repeated more than 100 times in different regions, and the normal force curves and histograms were obtained through normalization or statistics.

#### 3.1.4. Zeta Potential Measurements

Zeta potentials of asphaltene-brines and silica-brines interfaces were measured using a Zetasizer Nano (Nano-ZS, Malvern, UK) following the experimental method reported in previous literature [41]. Due to the fact the quartz crystal microbalance experiments were conducted at 25 °C, all zeta potential tests were conducted at the same temperature.

### 3.2. Surface Complexation Modelling

**Figure 5:**
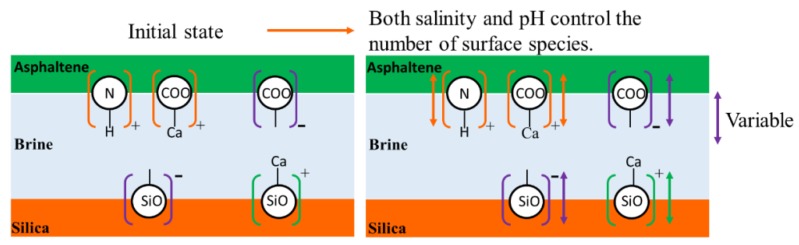
Schematic diagram of surface species in asphaltene-brine-silica system.

Given that asphaltene and silica surfaces are attached surface charges, surface complexation modelling appears to be a practical approach to gain a deeper understanding of the complex interaction of asphaltene-brine-silica system (Figure 5). We therefore performed a geochemical study and compared it with DLVO theory to delineate the interplay of asphaltene-brine-silica system. The surface species concentrations were calculated using PHREEQC version 3.3.9 (United States Geological Survey) in light of the diffuse layer surface model [42]. The geochemical reactions are given in Table 2 with equilibrium constants at 298 K. Note: in the surface complexation modelling, the acid number 2.75 mg KOH/g and base number, 12.34 mg KOH/g, were used as referring to the literature [33,43].

## 4. Implications and Conclusions

The low recovery of hydraulic fracturing fluid in unconventional reservoirs, such as tight sandstone, shale oil, and gas reservoirs, has received extensive attention both in terms of technology and environment in the last decade [3]. While a variety of mechanisms have been developed to understand why and how the fracturing fluids disappear, how the water chemistry affects the asphaltene-brine-silica system interplay and the surface wettability has received little attention at a molecular level. Here, the adsorption/desorption behavior and interaction forces of asphaltenes on the silica surface in the presence of different concentrations of NaCl were measured by QCM-D and CFM, respectively. Furthermore, we combined DLVO theory and surface complexation modelling to delineate the experimental results.

Our results showed that with the injection of water, there is a strong water uptake and the asphaltene adlayers become swelling, which may be one of the big challenges in tight sandstone reservoirs. Lowering salinity increases the asphaltene desorption and triggers a strongly negative zeta potential of both asphaltene-brine and brine-silica interfaces, implying a more water-wet system. We found that DLVO theory supports the experimental results, showing that decreasing salinity or increasing the pH value gives a bigger repulsive force which is confirmed by disjoining pressure calculation in this study and previous work. Surface complexation modelling further explains the increase of asphaltene desorption with decreasing salinity or increasing pH, which is attributed to less -NH^+^ sites at asphaltene-brine interfaces and lower number of bonds between asphaltene-brine and brine-silica interfaces. We conclude that hydraulic fracturing fluid increases the water-wetness, in favor of water uptake, while low salinity or high pH water facilitates the asphaltene desorption and inhibits the electrical double layer swelling, which in turn likely improves the water flowback in various applications.

## Figures and Tables

**Table 1:** Zeta potentials at different interfaces.

Concentration of NaCl Solution (mM)	Zeta Potential (mV)	
	Asphaltene-Brine	Brine-Silica
10	−17.1	−29.0
100	−12.8	−21.3
1000	−6.6	−1.5

**Table 2:** Surface complexation modelling parameters [44].

Geochemical Reactions		log K_298K_
Asphaltene surface	-NH^+^ = -N + H^+^	–6.0
	-COOH = -COO^–^ + H^+^	–5.0
Silica surface	>SiOH = >SiO^−^ + H^+^	–4.0 [44]

Note: As shown in Table 2, the “-” in -NH^+^ or -COOH represents the groups present at the asphaltene-brine interface [23], and “>” in >SiOH represents the groups present at the silica-brine interface [30,45].
